# Discovery of the potentially invasive Asian longhorned tick, *Haemaphysalis longicornis* Neumann (Acari: Ixodidae) in Türkiye: an unexpected finding through citizen science

**DOI:** 10.1007/s10493-025-01015-9

**Published:** 2025-04-09

**Authors:** Adem Keskin, Kandai Doi

**Affiliations:** 1 Department of Biology, Faculty of Science & Art, Tokat Gaziosmanpaşa University, Taşlıçiftlik, Tokat, 60250 Türkiye; 2 Department of Wildlife Biology, Forestry and Forest Products Research Institute, Matsunosato 1, Tsukuba, Ibaraki 305- 8687 Japan

**Keywords:** Human infestation, Invasive species, Parasite, Zoonotic diseases, Ticks

## Abstract

The Asian longhorned tick, *Haemaphysalis longicornis* Neumann, is a species commonly found in Central Asia, East Asia, and Australia, but it has recently emerged in the USA as a significant disease threat. The tick exhibits a fascinating biological trait, as certain populations are capable of reproducing both sexually and asexually via parthenogenesis. As a result, a single asexual female has the potential to initiate the establishment of a new population when introduced into a novel geographical region. *Haemaphysalis longicornis* is of considerable medical and veterinary importance, being associated with more than 30 human pathogens, including *Anaplasma*, *Babesia*, *Bartonella*, *Coxiella*, *Rickettsia*, *Theileria*, and others. It is also a competent vector for the severe fever with thrombocytopenia syndrome virus (Bunyaviridae, Phlebovirus) in Eastern Asia. The tick can also cause heavy infestations in cattle and transmit the hemoprotozoan parasite *Theileria orientalis* genotype Ikeda, resulting in significant economic losses within the cattle industry. In the present study, we report the morphological and molecular identification of *H. longicornis* in the European part of Türkiye. Additionally, we offer hypotheses regarding how *H. longicornis* ticks may have arrived in Türkiye, potential risks, and the necessary precautions that should be taken.

## Introduction

Invasive species are introduced to new environments where they can cause harm to local ecosystems. They can spread rapidly and outcompete or displace native species, and even disrupt ecosystems. Invasive species often thrive in new environments because they lack natural predators, diseases, or competitors that would limit their growth in their native habitats (Keller et al. 2011). The major reason for the invasion of species is human activity, such as global trade/transportation (ships and airplanes, or cargo etc.), habitat destruction/modification (urbanization, deforestation, agriculture etc.), climate change and intentional introduction (Hulme 2009; Keller et al. 2011; Pearson et al. 2022).

Blood-sucking arthropods, such as fleas, mosquitoes and ticks, can spread and establish themselves outside of their natural habitats, and they can become primary vectors of some pathogens for both animals and humans in non-native areas (Rogers and Randolph 2006; Cuthbert et al. 2023). The Asia-native *Rhipicephalus microplus* (Canestrini) and the Middle East and Mediterranean-native *Rhipicephalus annulatus* (Say) are some of the most important invasive ticks introduced to the southern United States and are responsible for the widespread invasion and spread of cattle babesiosis in this region (Giles et al. 2014; Okely and Al-Khalaf 2022). *Rhipicephalus microplus* has also been reported as the most successful invasive tick species in some Sub-Saharan African countries (Benin, Burkina Faso, Cameroon, Ivory Coast, Kenya, Nigeria, Mali and Togo) (Madder et al. 2011; Muhanguzi et al. 2020). In recent years, *Hyalomma marginatum* Koch and *Hyalomma rufipes* Koch, which were transported to central and northern Europe especially by migratory birds, are considered invasive species in Europe (Jameson et al. 2012; Estrada-Peña 2023).

The Asian longhorned tick, *Haemaphysalis longicornis* Neumann is native to eastern Asia, including Japan, China, Korea, and eastern Russia. Outside its natural range, *H. longicornis* was discovered in Australia in 1897. In the following years, this tick was detected in New Zealand, New Caledonia, and some Pacific islands (Vanuatu, Western Samoa, Fiji and Tonga) (Hoogstraal et al. 1968; Heath et al. 2011; Heath 2020). Although, retrospective studies revealed that *H. longicornis* were in the USA as early as 2010, it was reported for the first time in the USA in 2017 from New Jersey (Rainey et al. 2018). Subsequently, these invasive ticks were detected in over 20 jurisdictions, namely Arkansas, Connecticut, Delaware, Georgia, Illinois, Indiana, Kentucky, Maryland, Massachusetts, Missouri, New York, North Carolina, Ohio, Oklahoma, Pennsylvania, Rhode Island, South Carolina, Tennessee, Virginia and Washington DC (Egizi et al. 2019; USDA-APHIS-VS 2024; Myers and Scimeca 2024).

This study reports, for the first time, the presence of the highly invasive *H. longicornis* tick in Türkiye. Additionally, we provide suggestions regarding the potential pathways through which *H. longicornis* may have been introduced to Türkiye, its associated risks, and the necessary precautions that should be implemented.

## Materials and methods

### Morphological identification of ticks

In November 2024, a dog owner living in Istanbul metropolitan area reported to us that his dog had a serious tick infestation. Ticks collected by the owner between October and November 2024 were sent to the Parasitology Laboratory of the Department of Biology, Faculty of Science & Art, Tokat Gaziosmanpaşa University (Türkiye), for species identification. Ticks were examined under a stereomicroscope (Olympus SZ61, Olympus Corp., Tokyo, Japan) and identified using keys (Hoogstraal et al. 1968; Yamaguti et al. 1971; Filippova 1997). All ticks were deposited to the Parasitology Laboratory of the Department of Biology, Faculty of Science & Art, Tokat Gaziosmanpaşa University, Türkiye.

### DNA isolation, PCR and phylogenetic analyses

Total genomic DNA was isolated from a female engorged ticks according to the manufacturers’ protocol (PureLink™ Genomic DNA Mini Kit, Invitrogen, Carlsbad, CA, USA). DNA quantity and quality was quantified spectrophotometrically at 260/230 wavelength, with a microplate reader (Multiskan Go, Thermo Scientific, Vantaa, Finland). For the molecular identification, 16S rDNA gene region was amplified using 16S+1 (CTGCTCAATGATTTTTTAAATTGCTGTGG) and 16S-1(CCGGTCTGAACTCAGATCAAGT) primers (Black and Piesman 1994). PCR reaction was performed by using thermal cyclers (Bio-Rad T100 Thermal Cycler, Hercules, CA, USA). The content of the PCR reactions is 25 µl DreamTaq 2× PCR master mix (ThermoFisher Scientific, Waltham, MA, USA), 2 µl forward primer, 2 µl reverse primer, 2 µl template DNA and 19 µl DNA grade water. Thermal cycler conditions were as follows: 95 ^o^C for 5 min, then 40 cycles of 60 s at 94 ^o^C, 60 s at 51 ^o^C, and 45 s at 72 ^o^C, followed by 5 min at 72 ^o^C. The PCR products were run and visualized on 1% agarose gel electrophoresis. The PCR product, after purification, was sequenced bidirectionally by a commercial facility (Macrogen Inc., Amsterdam, Netherlands) using forward and reverse primers. The sequences were edited and aligned by using the BioEdit Sequence Alignment Editor, version 7.0.5 (Hall 1999). Phylogenetic analyses were performed by using MEGA 11 (Kumar et al. 2018).

## Results

**Fig. 1:**
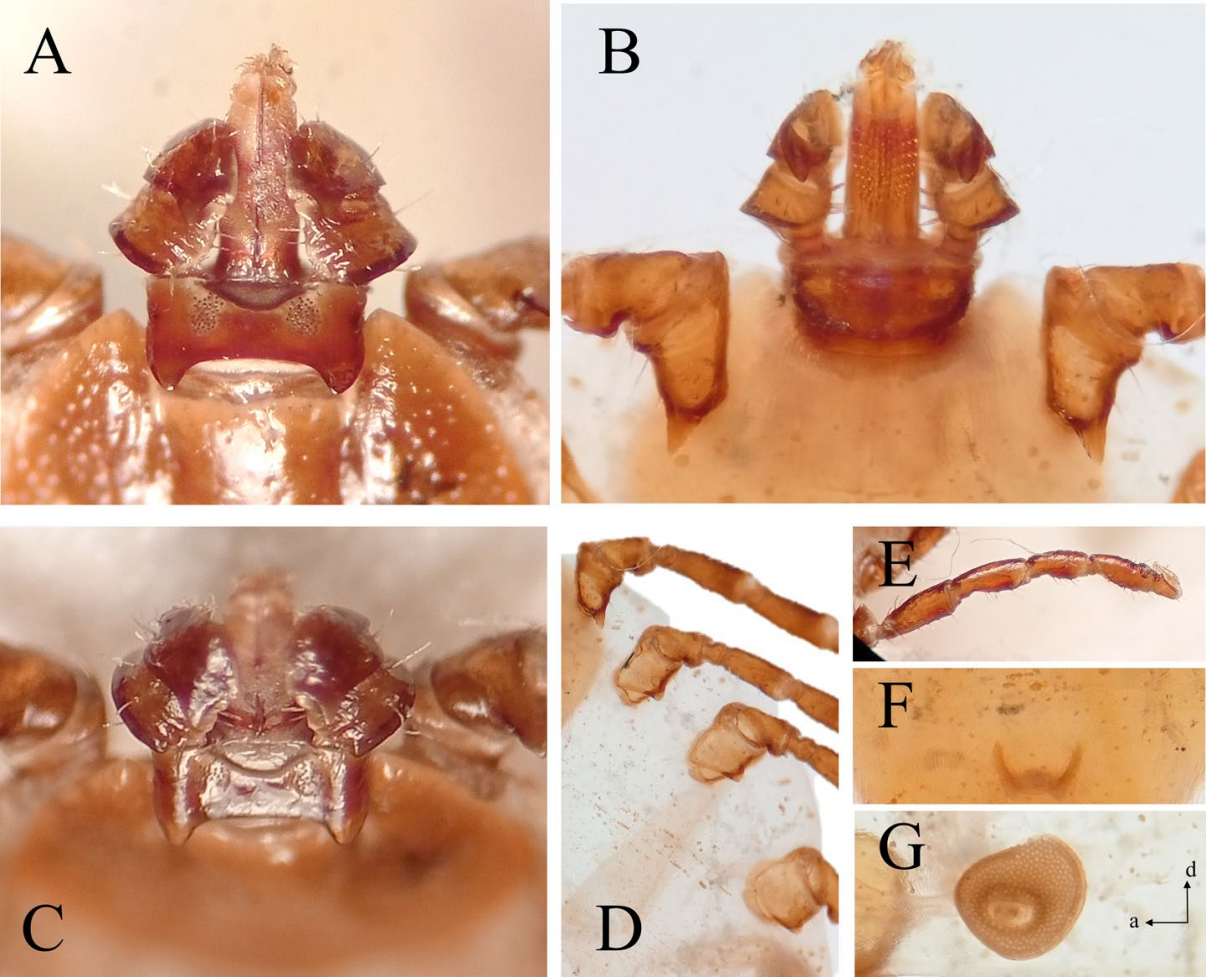
*Haemaphysalis longicornis* collected from domestic dog in Türkiye. **(A)** Gnathosoma, dorsal view; **(B)** Gnathosoma, ventral view and coxa I; **(C)** Spurs of palpal segment III, anterodorsal view; **(D)** Coxae; **(E)** First leg segments, **(F)** Genital opening, **(G)** Spiracular plate

Morphological examination of the ticks revealed that the majority belonged to the species *Ixodes ricinus* (L.) and *Rhipicephalus sanguineus* s.l. (Latreille), as anticipated. However, 9 female and 11 nymphal specimens were identified as belonging to the genus *Haemaphysalis*, specifically the subgenus *Kaiseriana* (a subgenus not currently recorded in the tick fauna of Turkish or even in Europe). Considering Türkiye’s geographical location on the migratory routes of birds traveling between Africa-Europe and Africa-Asia, we compared our samples with known *Kaiseriana* species (*Haemaphysalis aciculifer* Warburton, *Haemaphysalis rugose* Santos Dias, and *Haemaphysalis parmata* Neumann) found in Africa. Our samples, however, did not match with African *Kaiseriana* species. After further comparison with all known *Kaiseriana* species, we morphologically identified the specimens as *Haemaphysalis* (*Kaiseriana*) *longicornis* (Fig. 1). Subsequently, in December 2024, the dog owner collected additional ticks from his dog and sent them us for identification. This collection included numerous *I. ricinus* specimens, as well as another female *H. longicornis*.

The female *H. longicornis* can be distinguished from other *Haemaphysalis* species naturally occurring in Türkiye based on a combination of the following morphological characters: the laterally pointed second palpal segment and the distinct spur on the dorsal side of the third palpal segment, as well as a dental formula of 5/5. Nymphs of *H. longicornis* can be differentiated from other nymphal *Haemaphysalis* species found in Türkiye by the presence of well-developed spurs on the ventral side of the third palpal segment and coxa I, along with a dental formula of 3/3 (Hoogstraal et al. 1968; Yamaguti et al. 1971; Filippova 1997).

**Fig. 2:**
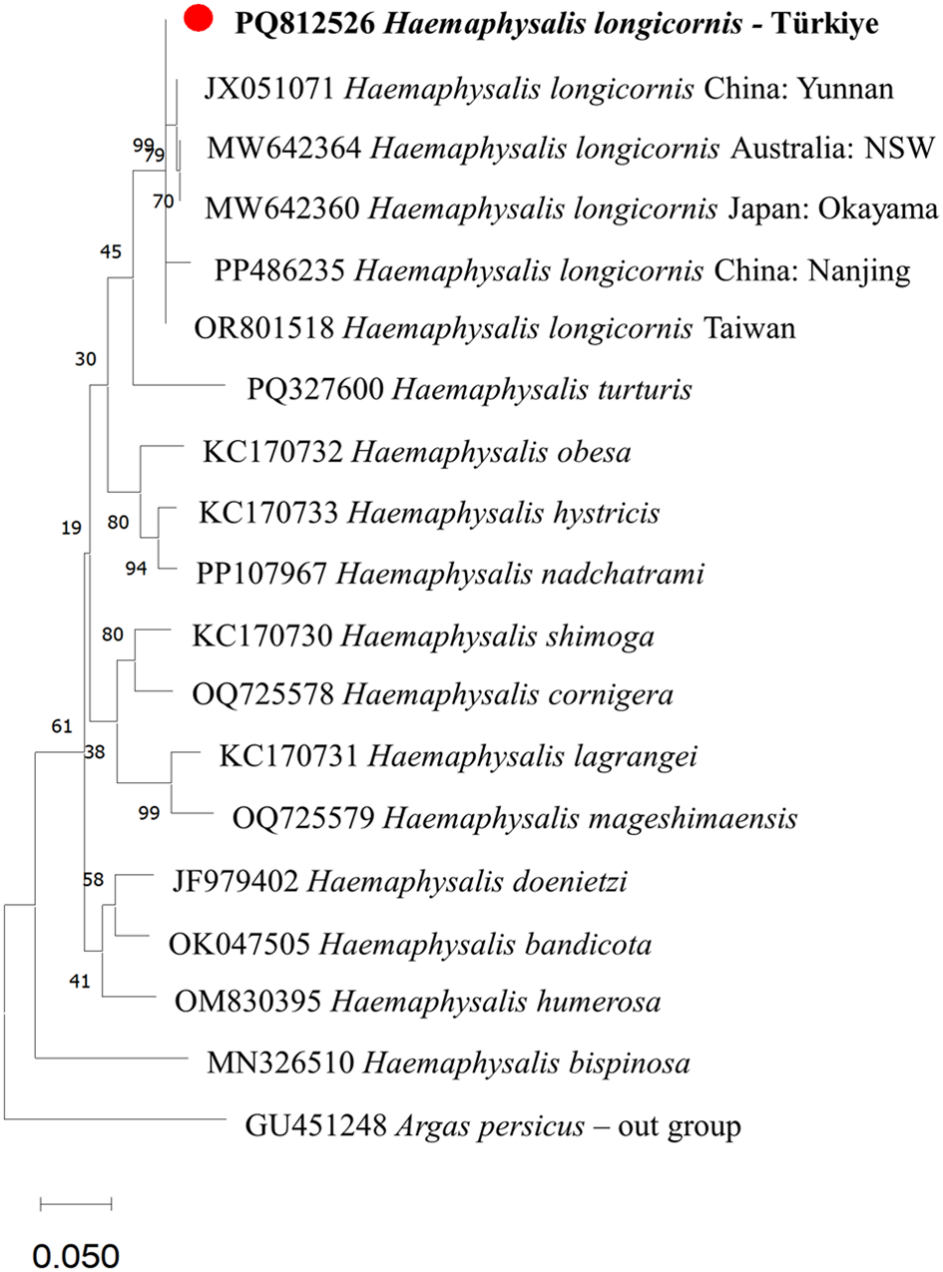
The phylogenetic tree was constructed based on partial 16S rRNA gene sequences of several species within the subgenus *Kaiseriana*

To accurately differentiate *H. longicornis* from other closely related species, such as *H. bispinosa* Neumann, and other species of the subgenus *Kaiseriana* (e.g., *H. aciculifer*, *H. rugose*, and *H. parmata*) found in the African region, molecular techniques are critical. Furthermore, molecular techniques are valuable for investigating the mechanisms through which this exotic species has invaded into Türkiye. Therefore, to confirm the identification of *H. longicornis*, molecular methods were also conducted. Sequence analysis of the 16S rRNA gene from our female *H. longicornis* specimen revealed a 99.78% sequence similarity to the *Haemaphysalis longicornis* isolate 23LH09-7-4 (accession number: PP486235) obtained from China (Nanjing). Furthermore, our isolate showed over 99% sequence similarity with other *H. longicornis* sequences from China (JX051064, MZ617267, PP486236, JX051069, and others). A phylogenetic tree was generated using maximum likelihood (ML) including the sequence of our isolate based on partial 16S rRNA gene sequences of some species of the subgenus *Kaiseriana* (Fig. 2).

## Discussion

The Asian longhorned tick has a wide host range. The main hosts of the tick are livestock hosts such as goats, sheep, cattle, and horses, but the tick can feed on a wide variety of wildlife including deer, rats, mice, hedgehogs, and birds (Heath 2016; Zhao et al. 2020; Okely et al. 2025). Sika deer (*Cervus nippon* Temminck) and other artiodactyls are frequently detected and immature stages are also detected from carnivores (Yano et al. 1988; Shimada et al. 2003; Zheng et al. 2012; Yamauchi et al. 2013; Tsukada et al. 2014; White et al. 2021).Wild birds, such as passerines, are also reported as hosts of the immature stages (Guglielmone et al. 2014). *H. longicornis* also infests humans from April to October (Inoue et al. 2020). *Haemaphysalis longicornis* is known to prefer grassland environments, like pasture, but they have also been collected along forest edges and within forests, indicating its tolerance to desiccation stress (Heath 1979; Schappach et al. 2020). This species is also known for its rapid transboundary spread as an invasive species. It has long been known to have expanded to Australia and New Zealand (Besier and Wroth 1985; Heath 2016). Egizi et al. (2020) claimed that *H. longicornis* has invaded North America and that its distribution will expand by 2024. One reason for the rapid expansion of *H. longicornis* is its parthenogenetic strain, which allows females to reproduce without mating. Compare to the bisexual strain in which males and females reproduce by copulation, the parthenogenetic strain is able to facilitate the establishment of new populations from single females. This strain has successfully invaded areas across the globe (Raghavan et al. 2019). There are concerns of migrating wild birds provided long distance dispersal of ticks. However, previous examples suggest that *H. longicornis* was likely transported into Oceania and North America with humans, luggage, or animals which traveled internationally. Companion animals travelling with humans may also act as long-distance dispersers (Egizi et al. 2020).

*Haemaphysalis longicornis* is known to harbor multiple pathogens including virus, bacteria, and protozoa. In Asian countries, *H. longicornis* is known as a vector of sever fever with thrombocytopenia syndrome (SFTS) virus (Oh et al. 2016). Also, *H. longicornis* is confirmed to have the ability to vector Heartland virus and Bourbon virus in the United States (Raney et al. 2022; Cumbie et al. 2022). *Rickettsia japonica*, the pathogen causes Japanese spotted fever is also frequently detected in *H. longicornis* (Tabara et al. 2011). In Australia, New Zealand, and North America, grazing cattle were infected with *Theileria orientalis* genotype Ikeda, the causative agent of bovine theileriosis, which is vectored by *H. longicornis* (Heath 2016; Thompson et al. 2020). The first case of *T. orientalis* Ikeda was recorded in Virginia, USA in 2017 and spread into 17 states in five years. This pathogen is causing major economic loss in the dairy farming industry in the USA (Thompson et al. 2020).

In this study, we detected *H. longicornis* in Türkiye for the first time. The molecular identification indicated that our samples are nearly identical to the parthenogenetic strain collected in China. The major concern is whether the parthenogenetic strain *H. longicornis* has already established the population in Türkiye. Invasion by the parthenogenetic strain of *H. longicornis* can often be rapid (e.g., in the US). Once *H. longicornis* is established in the natural environment, it will be difficult to eradicate. This is of particular concern given the prevalence of natural grazing of livestock in Türkiye. There is a possibility of protozoal infections, especially *T. orientalis*, in livestock and wildlife is of particular concerned. There is also a possibility of highly pathogenic viral diseases, like SFTS, being introduced from Asia. In addition, the Crimean-Congo hemorrhagic fever (CCHF) is endemic in Türkiye (Tekin et al. 2012; Leblebicioglu et al. 2014). The potential of *H. longicornis* to become a vector of CCHF virus is also of concerned as this could become a severe problem of public health in Türkiye.

Türkiye is strategically located at the intersection of Europe, Asia, and Africa. Consequently, various non-native tick species have been periodically reported in the country (Keskin and Erciyas-Yavuz 2016; Zerek et al. 2023). However, these reports are often incidental, and it remains unclear whether these ticks have established stable populations in Türkiye. Recent ecological niche modeling studies on *H. longicornis* have highlighted several potential habitat areas for this tick species in Europe, including the southeastern regions of Türkiye, under both current and future climate scenarios (Zhao et al. 2020; Okely et al. 2025). Therefore, it is crucial to examine the distribution of *H. longicornis* throughout Türkiye in relation to vegetation and host animals, as predicted by ecological niche modeling studies. Furthermore, there is an urgent need to investigate the potential pathogens associated with *H. longicornis* and evaluate its presence in domestic animals and wildlife. Such research is crucial for developing educational initiatives to mitigate public health risks and for implementing strategies to minimize the impact on the agricultural sector.

Finally, we aim to emphasize the importance of science to the general public, highlighting how citizen participation can substantially contribute to scientific progress and enhance the understanding of critical issues. Citizen science, which involves the active engagement of the public in scientific research, serves as a valuable tool that significantly broadens the scope and accelerates the pace of scientific projects by incorporating large numbers of individuals in data collection, observation, and analysis. This approach generates essential data that enables research to cover wider geographical areas, particularly in fields such as ecology, biodiversity, species distribution, and environmental sciences. Furthermore, citizen science increases public awareness of scientific matters and encourages greater involvement in scientific processes. The present study demonstrates the presence of *H. longicornis*, a potentially invasive species, in Türkiye, underscoring the critical role of citizen science in mapping the distribution of invasive species.

## Data Availability

The sequence data obtained from this study are freely available within the NCBI GenBank database under accession number PQ812526.
